# Gag-Positive Reservoir Cells Are Susceptible to HIV-Specific Cytotoxic T Lymphocyte Mediated Clearance *In Vitro* and Can Be Detected *In Vivo*

**DOI:** 10.1371/journal.pone.0071879

**Published:** 2013-08-07

**Authors:** Erin H. Graf, Matthew J. Pace, Bennett A. Peterson, Lindsay J. Lynch, Steve B. Chukwulebe, Angela M. Mexas, Farida Shaheen, Jeffrey N. Martin, Steven G. Deeks, Mark Connors, Stephen A. Migueles, Una O’Doherty

**Affiliations:** 1 Department of Pathology and Laboratory Medicine, University of Pennsylvania, Philadelphia, Pennsylvania, United States of America; 2 Laboratory of Immunoregulation, NIAID, National Institutes of Health, Bethesda, Maryland, United States of America; 3 The Center for Aids Research, University of Pennsylvania, Philadelphia, Pennsylvania, United States of America; 4 Department of Epidemiology and Biostatistics, University of California, San Francisco, San Francisco, California, United States of America; 5 Department of Medicine, University of California, San Francisco, San Francisco, California, United States of America; University of Alabama, United States of America

## Abstract

Resting CD4+ T cells infected with HIV persist in the presence of suppressive anti-viral therapy (ART) and are barriers to a cure. One potential curative approach, therapeutic vaccination, is fueled by recognition of the ability of a subset of elite controllers (EC) to control virus without therapy due to robust anti-HIV immune responses. Controllers have low levels of integrated HIV DNA and low levels of replication competent virus, suggesting a small reservoir. As our recent data indicates some reservoir cells can produce HIV proteins (termed GPR cells for Gag-positive reservoir cells), we hypothesized that a fraction of HIV-expressing resting CD4+ T cells could be efficiently targeted and cleared in individuals who control HIV via anti-HIV cytotoxic T lymphocytes (CTL). To test this we examined if superinfected resting CD4+ T cells from EC express HIV Gag without producing infectious virus and the susceptibility of these cells to CTL. We found that resting CD4+ T cells expressed HIV Gag and were cleared by autologous CD8+ T cells from EC. Importantly, we found the extent of CTL clearance in our *in vitro* assay correlates with in vivo reservoir size and that a population of Gag expressing resting CD4+ T cells exists *in vivo* in patients well controlled on therapy.

## Introduction

Resting CD4+ T cells harboring latent proviruses remain barriers to a cure for HIV [1–6]. As lifelong treatment with HAART is burdensome and cost prohibitive for many, strategies aimed at curing HIV are of great importance. While many strategies seek to clear reservoirs by stimulating HIV expression [7–11] recent data suggests that stimulation alone does not result in death of the infected cell [12]. Therefore, new approaches may be required to clear HIV reservoirs. Our group and others have turned to a unique population of HIV infected individuals who are able to control viremia without therapy [13–16] and appear to have smaller HIV reservoirs [17–19] (“elite controllers” or EC). Understanding how controllers maintain a low reservoir size may provide key insight into curing HIV [20].

We previously reported that CD4+ T cells from EC contain low levels of integrated HIV DNA (consistent with infrequent recovery of infectious virus [18,19]) and higher ratios of unintegrated to integrated HIV DNA when compared to individuals unable to control HIV (“non-controllers”) [17]. One major difference between these forms of HIV DNA is their ability to produce HIV proteins, as the unintegrated form is much less efficient at supporting HIV protein expression. Moreover, we recently reported, using an *in vitro* model of latency, that resting CD4+ T cells can produce HIV Gag without any stimulation and without producing infectious virus [21].

These prior observations, along with evidence that a subset of EC have a strong anti-Gag CD8+ T cell response associated with an overrepresentation of particular “protective” HLA class I alleles (B-57 and B-27) [22,23], led to our hypothesis that Gag-positive reservoir cells (GPR cells) are susceptible to cytotoxic T lymphocyte (CTL) mediated elimination in controllers, resulting in lower reservoir size. Therefore, we tested if EC-derived CD8+ T cells could clear GPR cells *in vitro*. To probe if reservoir size might be affected by CTL *in vivo* we compared reservoir levels to *in vitro* CTL activity and tested if GPR cells could be detected *in vivo*. Overall, our study provides important insight into how CTL might limit reservoir size.

## Results

### Superinfected resting CD4+ T cells from EC express HIV Gag without evidence of spreading infection suggesting that reservoir cells can express HIV Gag (Figure 1)

**Figure 1:**
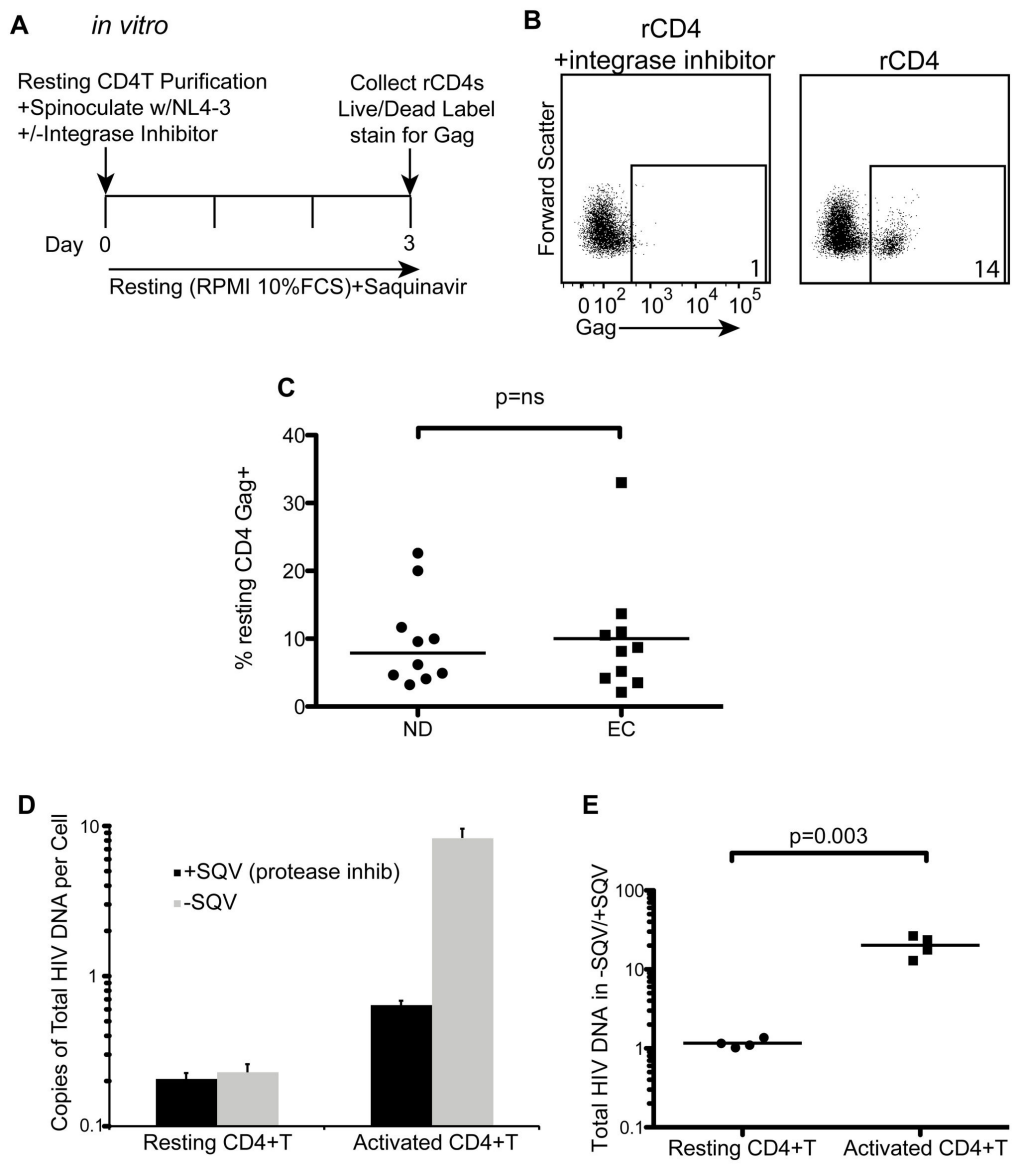
Superinfected resting CD4+ T cells express HIV proteins without producing detectable viral spread. (A) Resting CD4+ T cells were isolated from frozen PBMC by negative bead depletion and spinoculated with HIV-1_NL4-3_. Cells were cultured for 3 days without stimulation in the presence saquinavir (SQV). Resting conditions refer to culture in RPMI+ 10% FCS. After 3 days cells were collected, labeled with a viability dye and activation markers, then fixed and permeabilized and stained for intracellular HIV Gag. (B) Resting CD4+ T cells were gated by excluding HLA-DR, CD25 and CD69 and analyzed for the presence of intracellular Gag with or without addition of Raltegravir at the time of infection, using a reverse-transcriptase inhibitor-treated control also added at the time of infection to set the Gag positive gates. At time 0 and day 3 resting CD4+ T were determined to be 99% negative for activation markers (not shown) but consistent with [24]. (C) The frequency of cells expressing HIV Gag after *in vitro* infection and 3 day culture was compared among normal donors (ND, n=6) and elite controllers (EC, n=6). (D) Total HIV DNA was measured in superinfected resting or activated cells and compared between +SQV and -SQV fractions from one representative experiment in an EC. (E) Summary data from 4 different EC donors as in D, showing the ratio of total HIV DNA in the -SQV divided by the +SQV fraction. Lines represent the median and p values were generated using the Mann-Whitney test.

We recently showed that infected resting CD4+ T cells from normal donors can produce Gag without producing infectious virus [21]. We call these cells “Gag-positive reservoir cells” or GPR cells. Given the low *in vivo* levels of integrated HIV DNA in this cohort of EC [17], Gag expression would be challenging to detect directly *ex vivo*. Therefore, we tested whether resting CD4+ T cells from controllers superinfected with HIV-1_NL4-3_ could express Gag. Briefly, we isolated, spinoculated and cultured resting CD4+ T cells for 3 days to allow integration and protein expression (Figure 1A). Cells were labeled with a viability dye, stained for activation markers, then intracellularly stained for Gag. We found resting CD4+ T cells from EC and normal donors produce Gag without stimulation and this protein expression is reduced to near background levels upon addition of an integrase inhibitor (Figure 1B, n=6, p=0.01 compared to without integrase inhibitor).

We then wanted to examine whether Gag expression led to productive infection. We have previously shown in normal donors that Gag expression did not lead to the release of infectious virions in resting CD4+ T cells [21]. However, we had not studied the effect of superinfection on HIV infected individuals. We found that 99% of the resting cells lacked activation markers at time 0 and day 3 after superinfection (not shown), consistent with our previous results [24], suggesting superinfection and/or spinoculation does not result in activation. Additionally, we saw similar levels of Gag expression in normal donors and EC (Figure 1C), suggesting EC cells and normal donor cells are infected similarly [25]. To assess whether or not these cells could produce infectious virus, we inoculated resting and activated cells in the presence or absence of a protease inhibitor, as previously described [21], and measured total HIV DNA. If cells were productively infected, new rounds of replication should occur and total HIV DNA should increase in the untreated fraction. When comparing untreated to protease inhibitor treated cells, we saw a large increase in total HIV DNA in the controller-derived activated cells while we saw no increase in the controller-derived resting cells (Figure 1D). Four different EC were examined with similar and statistically significant differences in resting compared to activated cells (p=0.003, Figure 1E) with the resting cell fold change being close to 1, indicative of little to no spread and consistent with data from normal donors [21]. These results suggest that EC-derived GPR cells do not support detectable spreading infection.

### Gag expression in resting CD4+ T cells from EC is reduced with autologous CD8+ T cell coculture (Figure 2)

**Figure 2:**
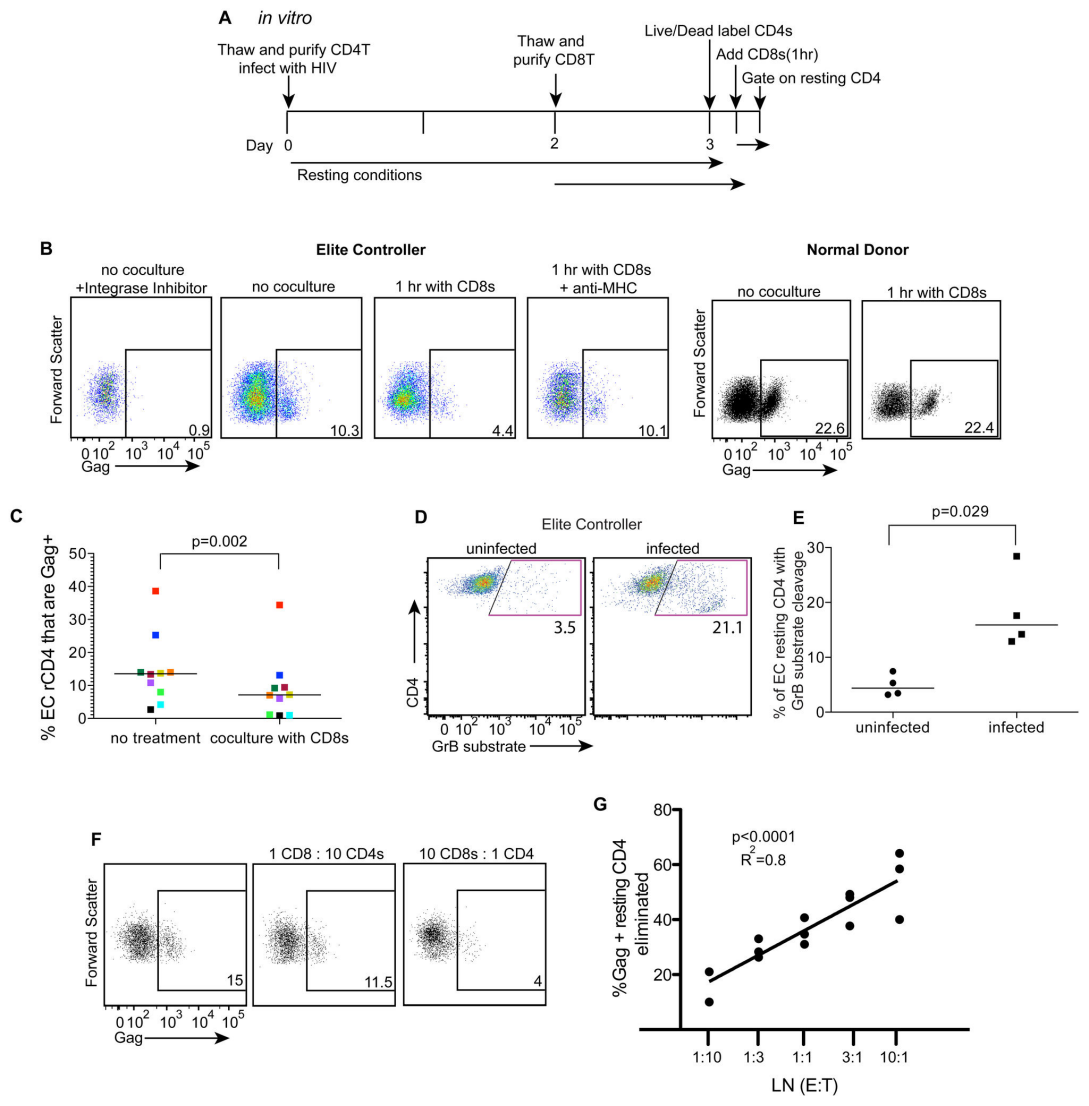
GPR cells from EC can be eliminated by autologous CD8+ T cells. (A) Resting CD4+ T cells were isolated from frozen PBMC and infected as in Figure 1. Autologous CD8+ T cells were purified from a second vial of frozen PBMC by negative bead depletion on day 2 and rested for 24 hours. After labeling the resting CD4+ T cell targets with a viability dye and activation markers, CD8+ T cells and CD4+ T cells were cocultured at a ratio of 10:1 for 1 hour. Cells were then fixed, permeabilized and stained for intracellular Gag and gated on activation marker negative CD4+ T cells for analysis. (B) A representative plot of gated resting CD4+ T cells from an EC or a normal donor that were either cultured alone for 1 hour (no coculture) or cocultured with CD8+ T cells for 1 hour (1hr with CD8s). The same cocultures were treated with an anti-MHC antibody or Raltegravir. The numbers represent the percent of cells that were positive for HIV Gag as above. (C) The percent of GPR cells after 3 days of culture post-infection was compared among samples either cultured alone or cultured with autologous CD8+ T cells for 1 hour (n=10). Lines represent the median and p values were calculated using a Wilcoxon matched-pairs signed rank test. (D) A representative plot of either uninfected or infected EC resting CD4+ T cells after loading with a granzyme B fluorescent substrate and coculture with autologous CD8+ T cells for 1 hour. The values represent the percent of cells positive for cleavage of the granzyme B substrate. (E) Uninfected and infected cells positive for cleavage of the granzyme B substrate were compared (n=4). Lines represent the median and p values were calculated using a Wilcoxon matched-pairs signed rank test. (F) A representative plot of EC resting CD4+ T cells infected with HIV, cultured for 3 days, and either cultured alone or cocultured with autologous CD8+ T cells at a ratio of 10:1 or 1:10 effectors to targets. The values represent the percent of cells positive for HIV Gag. (G) Combination of 3 experiments showing a linear correlation between the percent of infected cells eliminated as calculated by the reduction in HIV Gag positive cells and the natural log of the ratio of effectors to targets at 5 E:T ratios.

We questioned whether HIV Gag expression in resting CD4+ T cells is sufficient for recognition by CD8+ T cells and subsequent killing. To test this, we cocultured GPR cells from EC with autologous CD8+ T cells for 1 hour and measured a reduction in the percentage of GPR cells from the resting CD4+ T cell population [26]. Resting CD4+ T cells were isolated by negative selection, spinoculated with HIV, cultured for 3 days and then exposed to autologous, negatively selected, unprimed CD8+ T cells for 1 hour (Figure 2A). We found a reduction in GPR cells after coculture (Figure 2B). However, in a normal donor, there is no reduction (Figure 2B), as expected, since normal donors do not have sufficient HIV-specific CD8+ T cells. We compared frequency of GPR cells with and without CD8+ T cell coculture in ten different EC and saw a significant reduction in cocultured cells compared to non-cocultured controls (p<0.0001, Figure 2C). The reduction in GPR cells correlated with the effector to target ratio (r^2^=0.8 p<0.0001, Figure 2G).

To determine if the reduction in GPR cells was related to direct interaction between target resting CD4+ T cells and effector CD8+ T cells, we performed coculture experiments in the presence of an anti-MHC antibody (Figure 2B) or in a coculture assay with effectors and targets separated in a transwell plate (data not shown). In these experiments we saw little to no reduction in Gag expression. Thus, the reduction in Gag is likely MHC-I/T cell receptor contact dependent.

In order to test if elimination of resting CD4+ T cells is mediated by the granule exocytosis pathway, a major mechanism of CTL-mediated killing, we measured transfer of granzyme B from controller CD8+ T cell effectors to autologous resting CD4+ T cell targets after a 1-hour coculture using a previously described assay [26]. Briefly, CD4+ T cells are labeled with a viability dye, added to CD8+ T cells, and this mixture is loaded with a substrate for granzyme B. If lysis occurs via the granule exocytosis pathway, granzyme B will be transferred from the CD8+ T cells to target CD4+ T cells. Upon entering the target cell, granzyme B cleaves the previously loaded substrate, causing it to fluoresce. Thus, cells positive for transfer of granzyme B can be detected by flow cytometry. We found a significant increase in the percent of controller cells positive for transfer of granzyme B (Figure 2D,E) when coculturing infected resting targets compared with uninfected controls. We simultaneously found CD8+ T cells had enhanced expression of IFN-γ after coculture with autologous resting CD4+ T cell targets (not shown). Thus, CTL mediated killing of infected resting CD4+ T cells can occur via the granule exocytosis pathway suggesting an expanded target range greater than previously appreciated.

### Integrated HIV DNA Is Preferentially Cleared over 2-LTR Circles after Coculture Recreating the EC Phenotype and Is Not an Artifact of spinoculation (Figure 3)

**Figure 3:**
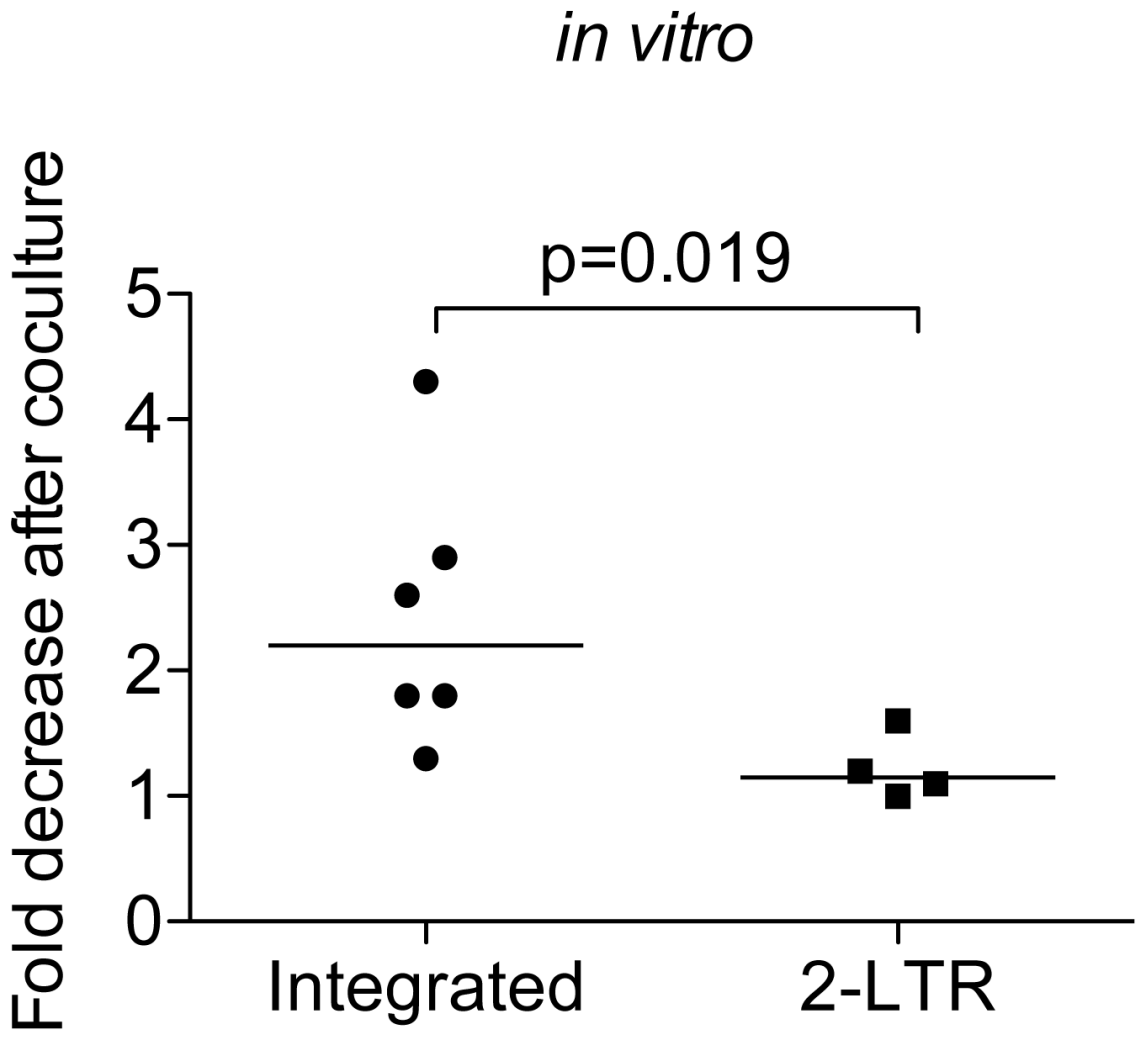
Integrated HIV DNA is preferentially cleared over 2-LTR circles after coculture providing an explanation of the EC phenotype of high levels of unintegrated relative to integrated HIV DNA; this clearance of integrated HIV DNA is not an artifact spinoculation. EC resting CD4+T cells were inoculated without spinoculation, cultured for 3 days and cocultured with autologous CD8+T cells or cultured alone for 16 hours. DNA was then isolated. The fold change is calculated by dividing the copies of the specific HIV DNA intermediate per resting CD4+T cell (i.e. integrated or 2-LTRs) cultured alone by the copies from the same samples cultured with autologous CD8+T cells. For cocultured samples the level measured was multiplied by 10, which is the dilution factor of effectors added to targets. The line represents the median and p value was calculated using the Mann-Whitney test.

We wanted to test whether or not we might be able to recreate the HIV DNA profile we recently reported in elite controllers [17] in our *in vitro* model. That is, we wanted to know if adding autologous EC CD8+ T cells to HIV superinfected resting CD4+ T cells would reduce integrated HIV DNA without reducing 2-LTR circular HIV DNA, since integrated HIV DNA but not 2-LTR circles expresses HIV Gag in an efficient manner (Figure 1B). In addition, we also wanted to perform experiments without spinoculation to confirm that our coculture results were not an artifact of this infection method. Since flow cytometry would not be sensitive enough to detect a reduction in HIV Gag expression without spinoculation, we indirectly tested if infected cells were eliminated under routine inoculation conditions by measuring DNA intermediates. If Gag expressing cells are cleared, we would expect that integrated HIV DNA would be reduced. On the other hand, most cells with unintegrated HIV DNA would be preserved since circles express HIV proteins much less frequently than integrated HIV DNA (Figure 1B and [27]). To test this, we compared the difference in the reduction of integrated versus 2-LTR circular HIV DNA in our coculture assay. We saw a statistically greater reduction in integrated compared to 2-LTR circular HIV DNA (Figure 3) after adding CD8+ T cells (10:1 E: T). These results suggest that Gag expressing cells are preferentially cleared. Importantly, they also suggest that the reduction in Gag is not an artifact of spinoculation, consistent with our prior data showing Gag expression without spinoculation [21]. These data are also consistent with our *in vivo* findings that controllers have extremely low levels of integrated HIV DNA and high levels of unintegrated, often 2-LTR circular, HIV DNA [17].

### GPR cells are more efficiently cleared by CTL in EC than in non-controllers (Figure 4)

**Figure 4:**
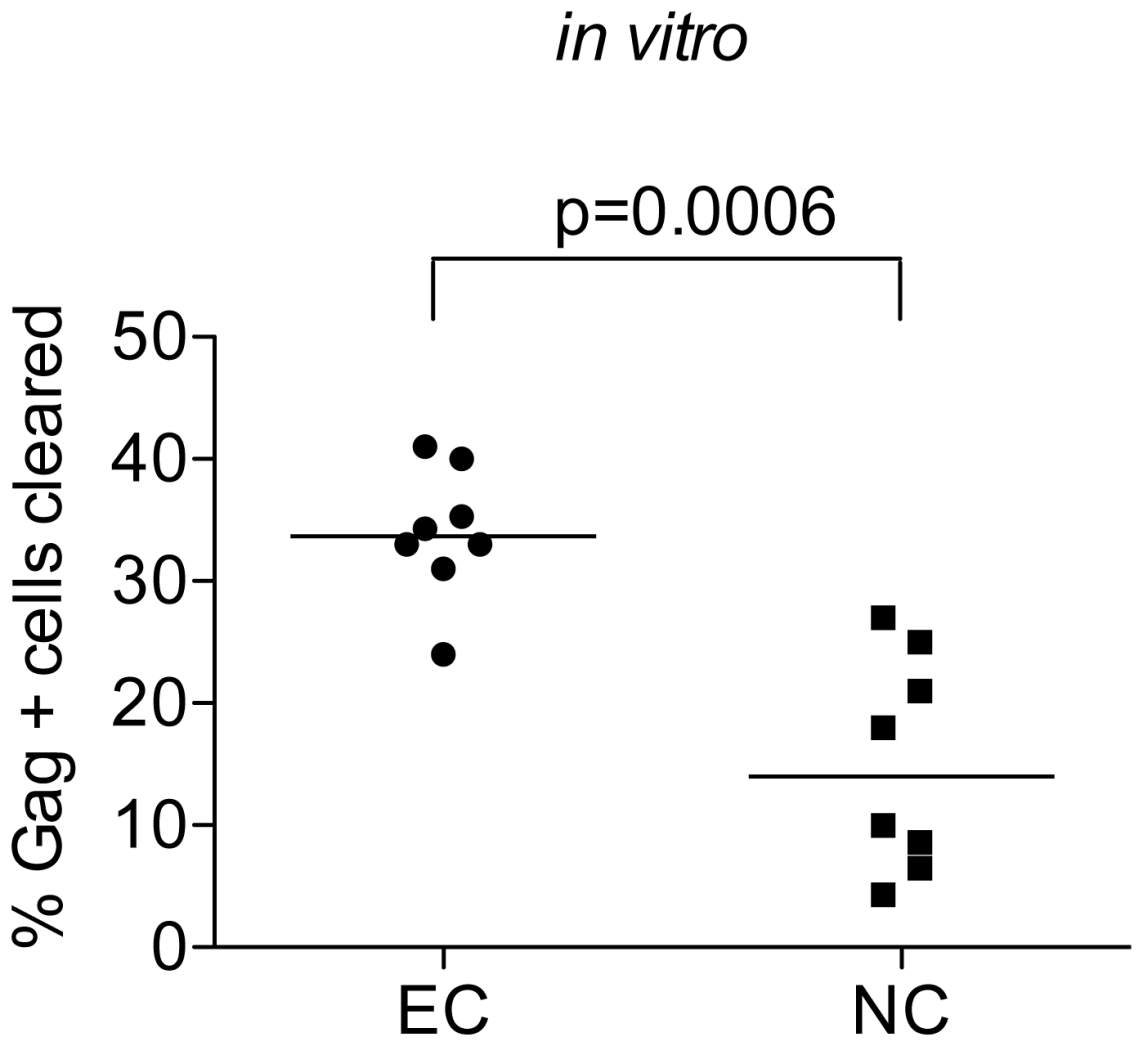
GPR cells are more efficiently cleared by CTL in EC than in non-controllers The percent of GPR cells eliminated after 1 hour coculture with autologous CD8+T cells was compared between EC (n=8) and chronic non-controllers (n=8). The line represents the median and the p value was calculated by a Mann-Whitney test.

If CTL are responsible for the smaller reservoirs found *in vivo* in controllers compared to chronically infected non-controllers [17] we would expect larger CTL clearance of GPR cells in EC than in non-controllers. Indeed, there was a significantly greater reduction of GPR cells by CD8+ T cells from controllers compared to non-controllers (Figure 4B, p=0.0001). This suggests either that controller-derived CTL are better able to target and remove GPR cells or that controller-derived GPR cells are more susceptible to CTL-mediated lysis. Given previous studies, our results are likely due to more effective CTL [23,26,28,29]. Additionally, our data are not due to a difference in the frequency of HIV specific CD8+ T cells as our EC and non-controllers had similar levels of HIV specific CD8+ T cells (data not shown).

### CTL clearance of GPR cells correlates with reservoir size in vivo (Figure 5)

**Figure 5:**
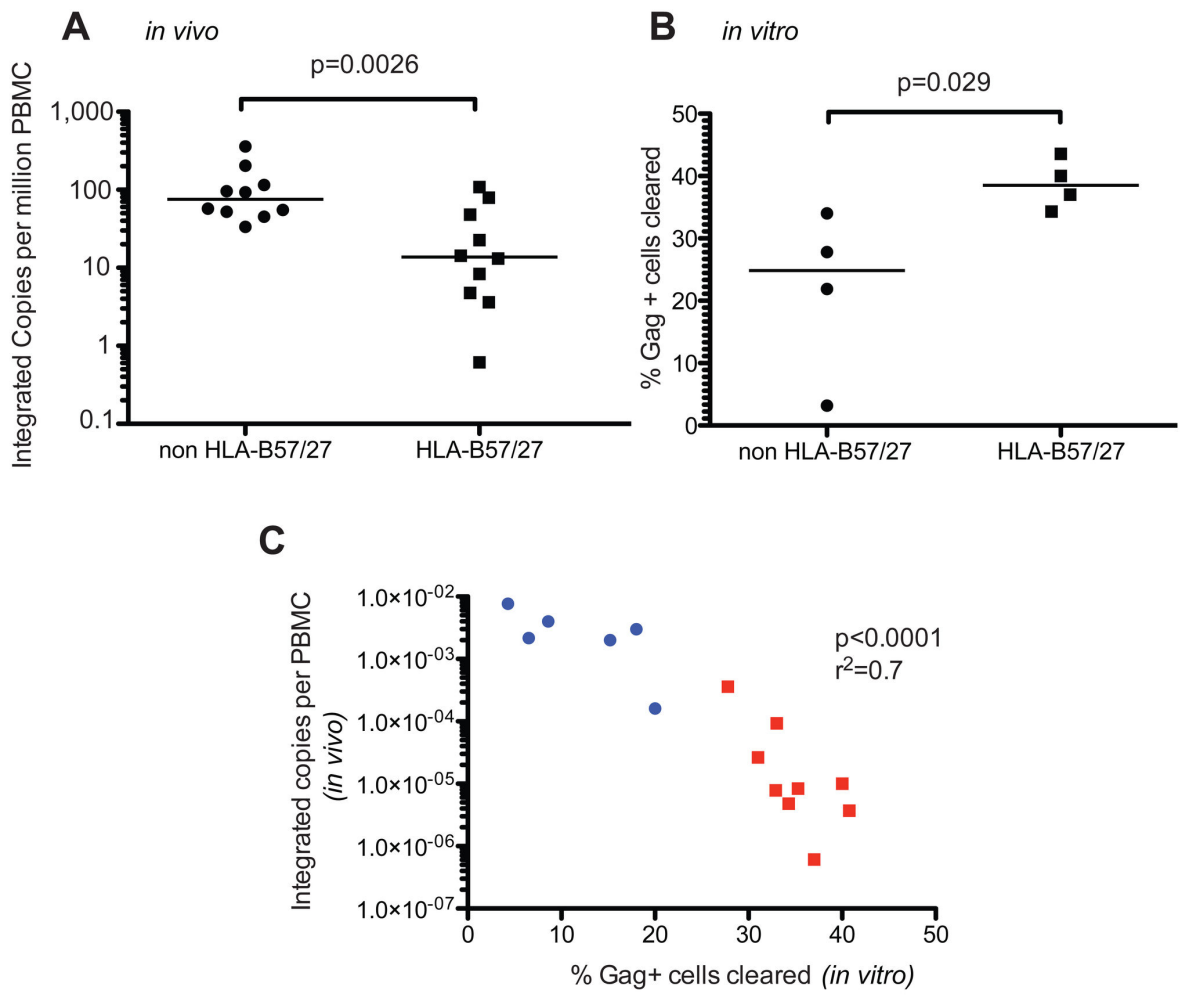
CTL clearance of GPR cells correlates with reservoir size *in vivo*. (A) Integrated copies per PBMC are plotted for EC with either B57 or B27 or EC without either allele. The line represents the median and the p value was calculated by a Mann-Whitney test. (B) A subset of the patients measured in A was assayed for killing ability as in Figure 4 (n=4 for either HLA group). The line represents the median and the p value was calculated by a Mann-Whitney test. (C) Percent of GPR cells eliminated (as in Figure 4B) after one-hour coculture with autologous CD8+T cells (at 1:1 E:T) is plotted on the x-axis. Integrated copies per million PBMC are plotted for the same patients. EC are represented by red squares and non-controllers by blue circles. The linear correlation was examined using the Pearson test with the p and r^2^ values reported (n=15).

We further tested our hypothesis that CTL control the level of integrated HIV DNA by correlating integration levels to activity against GPR. Although we have previously shown that chronic non-controllers have more integrated HIV DNA than EC [17], this could be attributed to differences in viremia, viral potency, or activation status. Therefore, we decided to examine integrated HIV DNA levels in two distinct cohorts of EC: those who express HLA-B57 or HLA-B27 and hence are likely controlling virus in part through CTL [22,28] and those who do not express these protective alleles and hence may be controlling virus though alternative mechanisms [22,30]. We measured the levels of integrated HIV DNA in PBMC from ten EC with either HLA-B57 or B-27 and ten EC without these alleles. Consistent with our hypothesis, we found a significantly lower level of integrated HIV DNA in the B57/27 cohort (Figure 5A, p=0.0023) suggesting the presence of these alleles is associated with more effective clearance of the reservoir.

Though the B57/27 alleles are associated with HIV control, there is no direct evidence to support better CTL function against GPR in EC with these alleles compared to EC without them. Thus, to test whether HLA-B57 or B27 possessing EC have stronger anti-HIV CTL, we performed the *in vitro* GPR clearance experiments in a subset of the patients measured in Figure 5A. We found that CD8+ T cells from EC with protective alleles were better at clearing GPR cells than EC without these alleles (Figure 5B, p=0.014). Thus EC with higher CTL activity against GPR had lower reservoirs.

We wanted to further test our hypothesis that CTL can control reservoir size *in vivo* by directly correlating *in vivo* reservoir size with the percentage of GPR cells cleared in our CTL assay. We studied a number of HIV infected individuals with a wide range of integration levels from 1 copy per million PBMC up to almost 1 copy per 100 PBMC. These individuals were a combination of chronic non-controllers and EC. We found a significant linear correlation between *in vivo* reservoir size and clearance of GPR cells *in vitro* (Figure 5C, p<0.0001). When plotted individually both the EC (red squares) and non-controller (blue circles) correlations reached significance as well (p=0.02 and p=0.048, respectively). These results suggest that CTL may clear GPR cells *in vivo* and thus may in part control reservoir size.

### GPR cells are detectable *in vivo* (Figure 6)

**Figure 6:**
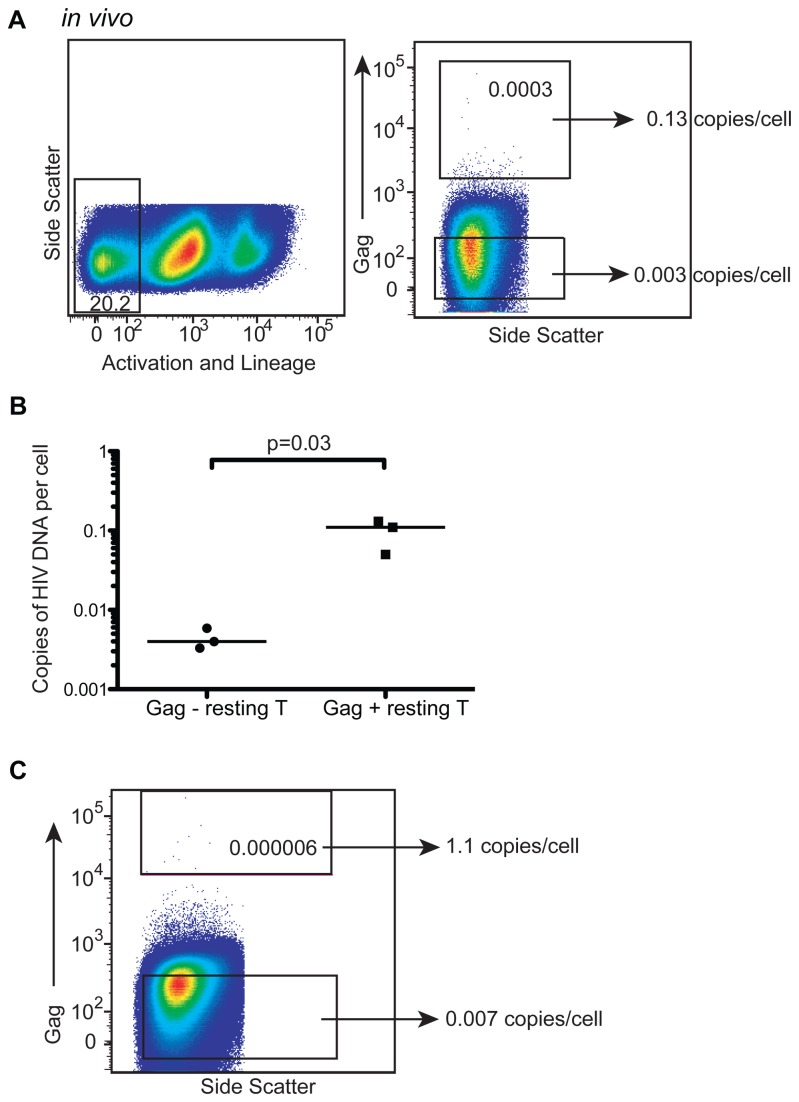
GPR cells are detectable *in vivo*. (A) Representative sort plots and gating strategies from a treated non-controller showing selection of resting CD4+T cells by lineage (except CD4) and activation markers stained in PE (left plot) and the selection of Gag positive and negative cells (right plot). The numbers inside the boxes represent proportion of resting CD4+T cells. Numbers outside of the boxes represent the copies of HIV DNA per cell. TP1 in Table 1. (B) Summary data from 3 sort experiments where HIV DNA was measured in Gag negative and Gag positive sorted populations from ART-suppressed non-controllers as in (A). A one-tailed paired Student’s t-test was used to determine statistical significance. We chose a one-tailed test because a priori we expected that sorted Gag positive cells would be enriched for HIV DNA. If for example, the staining were entirely nonspecific which is the alternative to our hypothesis, we would expect that there would be no enrichment of HIV DNA. There is no rationale for why the sorted Gag positive cells would be depleted for HIV DNA. Lines represent median values. (C) Sort data from one individual was gated more conservatively than the 3 previous sorts for Gag positive cells. The numbers inside the boxes represent proportion of resting CD4+T cells. Numbers outside of the boxes represent the copies of HIV DNA per cell. TP3 in Table 1.

Ultimately, we wanted to determine if GPR cells exist *in vivo* as this would suggest that a subset of the infected resting CD4+ T cell reservoir can be CTL targets. Given that the frequency of cells containing integrated HIV DNA is low in patients on ART, this is a difficult task. We approached the question by staining PBMC from ART-treated patients and sorted Gag-positive and negative resting CD4+ T cells (Figure 6A). We hypothesized that if any of the staining were specific for HIV Gag, we would see an enrichment of HIV DNA in these cells after sorting them. Consistent with our hypothesis we were able to detect a significant enrichment of HIV DNA in the Gag-positive resting CD4+ T cells compared to Gag-negative resting CD4+ T cells in 3 different donors (Figure 6B, p=0.03). However, the frequency of HIV DNA in the Gag+ cells was less than one, indicating that there was a component of nonspecific staining. To address the issue of nonspecific staining, in a separate experiment using more conservative gating for Gag, we were able to detect ~1 copy of HIV DNA per cell (Figure 6C, 100% infected). This suggested the strongly stained cells were truly positive for Gag expression and were not the result of non-specific binding. We were able to repeat this experiment in another donor with similar results (data not shown). Overall, our data indicates that GPR cells exist *in vivo* in patients on HAART.

As we had previously measured integration levels in these ART-treated non-controllers [31] we were able to calculate the percentage of integrated HIV DNA expressing HIV Gag *in vivo*. This ranged from 0.1 to 2 percent Gag expression per integrated copy (Table 1). Thus, only a small fraction of the reservoir is expressed at any given moment. However, what fraction of the reservoir is capable of being spontaneously expressed remains to be determined.

**Table 1:** HIV Gag is expressed *in vivo* in resting CD4+ T cells.

Patient	Years VL<50	Gag+/all sorted cells	Correction for live cells	Integrated HIV/PBMC	RU5/rCD4	Gag+/INT (%)
TP1	3	0.0000083	0.877	0.000073	0.131	2%
TP2	4	0.0000313	0.3524	0.0018	0.052	0.3%
TP3	2	0.0000010	0.975	0.00082	1.143	0.1%
TP4	2	0.0000027	0.986	0.00133	2.163	0.4%

The percentage of integrated HIV expressing HIV Gag was calculated by dividing the Gag positive cells per sorted cells (column 3) by the correction for live cells (column 4). We then divided this number by the integrated copies per PBMC (column 5) and multiplied by the RU5 per cell of the sorted Gag + resting CD4+T cells (and then multiplied by 100) to get the percent of integrated expressing Gag. This calculation assumes that all of the integrated HIV DNA is in the resting CD4+ T cell compartment of the PBMC, which makes the percent expressed an underestimate. Notably, we also measured the HIV DNA in the sorted Gag+ and Gag- fractions and were able to find a similar estimate of the frequency of Gag+ cells. In the case of 6A, which is TP1, we divided the amount of HIV DNA measured in the sorted Gag+ resting CD4+ T cells (HIV DNA copies per cell multiplied by the frequency of positive events) by the amount of HIV DNA measured in the sorted Gag- resting CD4+ T cells (HIV DNA copies per cell), and we obtained a similar frequency of Gag expression as calculated in Table 1. For example, 0.13 copies/cell x 0.0003 frequency of Gag+ cells/0.003 copies per cell in the Gag- fraction = 1.3% which is similar to the estimate in Table 1.

## Discussion

A new emphasis has recently been placed on curing HIV and many studies have focused on EC in the hopes they can provide insight into developing therapies allowing others to control infection. However, little attention has been paid to the role the immune response may play in limiting the HIV reservoir in infected individuals. Here, we show for the first time that effective CTL can clear resting CD4+ T cells expressing Gag (termed Gag-positive reservoir cells or GPR cells) *in vitro* and that this ability correlates with *in vivo* reservoir size. Importantly, even non-controllers well suppressed on ART have GPR cells detectable *in vivo* that could be targeted by the immune system, even without other activating agents such as vorinostat. These results suggest both that CTL may play an important role in controlling reservoir size *in vivo* and that it may be possible to harness adaptive responses to accelerate the decay of the reservoir in long-term ART treated patients.

While many previous studies have examined CTL activity against infected cells *in vitro*, these studies artificially stimulated CD4+ T cells and/or CD8+ T cells, potentially altering HIV protein expression and killing effectiveness [12,26,32] or assumed only the activated portion of unstimulated CD4^+^ T cells expressed HIV [25,33]. Here, we found unstimulated CD8^+^ T cells that likely represent effectors *in vivo* could clear resting CD4+ T cells expressing Gag, at least in part through the granule exocytosis pathway. We could not detect spreading infection in these resting cells, suggesting HIV-protein expression alone was sufficient for clearance. While some studies have suggested spinoculated cells may not reflect latency *in vivo*, our previous work showed that spinoculation did not affect the viral life cycle beyond binding and was not required for Gag production [21]. Furthermore, spinoculation was not required to see the clearance of infected resting CD4+ T cells as shown by the clearance of integrated HIV DNA integration measurements. Thus, spinoculation of unactivated CD4^+^ T cells can provide a useful model for CTL clearance and their utility have been recently confirmed by others [25,33,34]. Overall, our experiments suggest that native CTL in both EC and to a much lesser extent chronic non-controllers may be able to target a population of resting CD4+ T cells that produce Gag.

We additionally pursued how our data reflected our prior *in vivo* findings that EC had very low levels of integrated HIV DNA but relatively high levels of unintegrated DNA, particularly 2-LTR circles [17]. Consistent with this finding, integrated HIV DNA was more efficiently cleared in our coculture assays than 2-LTR circles suggesting CTL may explain the unique DNA profile in EC. It remains possible that other mechanisms besides CTL activity contribute to this DNA profile, such as a block to integration as recently proposed [35]. However, in our system we find integration occurs with similar efficiency in EC and normal donors [17]. We acknowledge that EC are a heterogeneous population such that different mechanisms may apply to different cohorts.

As several explanations besides CTL activity could explain small reservoirs in EC we compared integration levels in two different cohorts of EC: those with HLA-B57 or HLA-B27 and those without those alleles. There is evidence that CD8+ T cells from EC with protective alleles have stronger Gag-specific responses than EC without these alleles [30]. We indeed found that EC with HLA-B57 or HLA-B27 had more effective CTL activity in our coculture assay and had lower reservoirs. This data is consistent with studies showing that effective Gag epitopes are important in controlling HIV [36,37]. Interestingly, this data is also consistent with our *in vitro* data that GPR cells can express Gag but not Env [21], as clearance by EC with effective Gag epitopes and greater Gag clearance had lower reservoirs.

This finding was not restricted to EC. When we examined 15 patients (a combination of EC and non-controllers), we found a strong correlation between CTL activity *in vitro* and *in vivo* reservoir size. Importantly, both EC and non-controllers correlated if graphed separately, suggesting the correlation was not due to the extremely low reservoirs of EC. Overall, these *in vivo* data suggest that CTL activity may control reservoir size.

Our previous studies showed resting CD4+ T cells could express Gag *in vitro* [21]. Prior studies in HAART treated non-controllers showed HIV RNA expression could occur even in resting CD4+ T cells [38–42], but given the inefficiency of HIV RNA export in resting CD4+ T cells [42], it remained unclear if resting CD4+ T cells expressed HIV Gag in vivo. Here, we show for the first time that a population of resting CD4+ T cells in HAART treated non-controllers express Gag *in vivo*. This Gag staining was not due to non-specific binding as the Gag positive cells were enriched for HIV DNA.

We have refrained from calling GPR cells latent although the lack of productive infection (Figure 1) and [21] suggest that they appear to be latently infected. We realize that many would consider a truly latent cell to be transcriptionally silent. Therefore we have chosen the term “Gag-positive reservoir cell” to define this population. As these patients examined for GPR cells *in vivo* were well controlled on HAART for at least 2 years, the Gag production reflects a portion of the reservoir that is expressed and resistant to anti-retroviral therapy. Regardless of whether or not these cells are latent, GPR cells represent a potential target for the immune system.

Our results have clear implications for therapeutic design. As GPR cells can be targeted by CTL, therapeutically enhancing the immune system may allow the clearance of these cells, which would potentially reduce the reservoir. Our findings that an effective CTL response might reduce the size of the reservoir in the absence of HIV replication is broadly consistent with a recent study that found an inverse correlation between mucosal HIV-specific T cells responses and the frequency of HIV DNA-containing PBMC [43] as well as the findings that effective vaccination in non-human primates [44] and therapeutic vaccination in humans [45] appeared to block establishment of or reduce the latent reservoir, respectively.

On the whole, our data indicate the importance of CTL in controlling HIV reservoirs. While it seems unlikely that HIV can be cured by enhanced CTL alone, our data suggest it can be dramatically reduced. Thus, efforts to boost the immune response should be pursued not only to control infection but in conjunction with other efforts to enhance HIV expression in order to cure HIV.

## Experimental Procedures

### Ethics Statement and study subjects

EC and untreated/chronically infected subjects were recruited from the Clinical Reasearch Center, NIH (Bethesda), the Center for Aids Research (CFAR) at the University of Pennsylvania or the SCOPE cohort at the University of California, San Francisco (UCSF). All participants signed informed consent forms approved by the NIAID IRB, the University of Pennsylvania’s IRB and UCSF’s IRB. The University of Pennsylvania IRB approved the transfer of materials from NIH and UCSF. The EC used for *in vitro* studies were previously described [17]. The chronically infected non-controllers were also previously described [26]. PBMC from normal donors were obtained through anonymous donation to the University of Pennsylvania’s Human Immunology Core.

### Isolation of resting CD4+ T cells

Frozen PBMC were thawed and resting CD4+ T cells were negatively selected by depletion of lineage markers CD20, CD16, CD14, CD56, CD8, BDCA2, CD11c, and activation markers (HLA-DR, CD69 and CD25) following the MACS LD depletion protocol (Miltenyi). Resting CD4+ T cells were >99% pure.

### Inoculation and Gag staining

Transfection supernatants were prepared by the Penn CFAR using HIV-1_NL4-3_. Spinoculation, with virus at an MOI of 3, was used for Figures 1, 2, and 4 in order to ensure Gag signal easily detectable above background [24,46]. Resting CD4+ T cells were spinoculated as previously described in the presence of 8µg/mL polybrene for 2h [21]. Cells were then washed and treated with 50µg/mL Dnase 1 (Roche) and 10mM MgCl_2_ for 2h. Cells were then washed and cultured in RPMI+10% FCS with 1.25 µM saquinavir (SQV) (Roche) and with or without 100nM Raltegravir (Merck) for 3 days. Cells were harvested at 72h post-infection and labeled with a viability dye in Pacific Blue (LIVE/DEAD, Invitrogen) following the manufacturer’s instructions. Cells were then labeled with CD8-PE, CD3-PerCP, and activation markers- APC (Miltenyi). Following the FIX & PERM kit protocol (Invitrogen), cells were fixed and permeabilized while exposed to KC57-FITC antibody (Beckman Coulter) for intracellular Gag staining. Data was collected on an LSR II (BD Biosciences) and analyzed with gates set on resting CD4+ T cells before Gag analysis. Matched integrase inhibitor controls were used to set gates for Gag expression to keep the background between 0.5–1% as in [47].

### Spreading infection

Resting CD4+ T cells from EC isolated as above were cultured for 3 days in RPMI+10% FCS alone or with anti-CD3/CD28 beads (Invitrogen) at a concentration of 3 beads/cell + 100U/mL IL-2 (R&D Biosystems). Afterwards, cells were washed, spinoculated and DNase treated as above. Half of each sample (resting or activated) were treated with 1.25µM SQV. Cells were collected (3 days post infection for activated and 7 days for resting cells. DNA was prepared using the QIAamp DNA Micro Kit (Qiagen). Cells per well were quantified by real-time PCR for the β-Globin gene and total HIV DNA was measured by real-time PCR as in (33).

### Coculture experiments

CD8+ T cells were negatively selected from a separate frozen aliquot of PBMC by depletion of lineage markers CD20, CD16, CD14, CD56, CD4, BDCA2 and CD11c following the MACS LD column depletion protocol (Miltenyi). After overnight culture in RPMI+10% FCS, CD8+ T cells were harvested and counted. Simultaneously, 72h post infection resting CD4+ T cells were also harvested, stained with LIVE/DEAD cell viability dye and counted. Cells were combined at the appropriate E:T ratio (e.g. 10e6 CD8+ T cells were combined with 1e6 CD4+ T cells for a 10:1 ratio). The combined cells were spun down, resuspended in RPMI+10% FCS to a concentration of 1e6 cells/ 100µL, plated and incubated for 1h at 37°C. After 1h, cells were harvested and labeled and intracellularly stained as above.

### Granzyme B transfer assay

Infection and coculture conditions were as above except the E:T ratio was 25:1 as in [26]. After addition of targets and effectors, the combined cells were resuspended in Granzyme B substrate (OncoImmunin, Inc.) for a 1-h incubation. After coculture, samples were washed and analyzed by flow cytometry. The viability dye separated targets from effectors as well as live from dead cells. The substrate was picked up in the FITC channel upon cleavage.

### Non-spinoculated experiments

For HIV DNA intermediate experiments, the same virus was used as described above without spinoculation or polybrene. Rather, the cells were exposed to virus for 2 h at 25°C, washed, DNase treated as above and then cultured at 37°C. These samples were harvested 72 hours post-infection and cocultured as above. All non-spinoculated experiments were performed at an E:T ratio of 10:1. In these experiments, cells were cocultured for 16 hours before harvesting. The DNA was isolated using the Qiamp DNA micro kit (Qiagen).

### PCR measurements

Integrated HIV DNA in superinfected samples was measured as in [48]. 7,500 cells were assayed per well with 10 repeats. A standard curve on each plate was used to calculate the copies of integrated HIV DNA per cell. 2-LTR circles were measured as reported [17]. Cells were assayed at 1e5, 5e4 and 2.5e4 per well in triplicate and the average is reported. An albumin quantitative PCR was used to determine cell quantity as described [17]. Integration and 2-LTR copies per cell from cocultured samples were multiplied by 10 to account for the dilution factor of effectors to targets.

### HLA-B57/27 and non-B57/27 EC measurements

Frozen PBMC were obtained from the SCOPE study cohort at UCSF. 10e6 cells per patient were isolated using the Blood and Cell Culture DNA Midi Kit (Qiagen). Genome concentrations were calculated using an Albumin assay as described [17]. Integration was measured as in [17]. EC were grouped in those having the HLA-B57 or B-27 allele and those without. CD4 and CD8 counts were not statistically different between the groups.

### Sorting experiments

Frozen PBMC from patients well suppressed on ART were thawed and stained with the following lineage markers in PE: CD8, CD14, CD16, CD20, BDCA2, CD11c, CD56, and activation markers in APC. These cells were then stained for intracellular Gag by fixation with 0.5% paraformaldehyde and permabilization with 0.5% Saponin (Sigma) and stained with KC57-FITC (Beckman Coulter). We sorted CD3+, lineage negative (except CD4) cells, which lacked activation markers. We then sorted both Gag positive and Gag negative resting CD4+ T cells. Total cellular DNA was isolated from sorted cells using the DNeasy mini kit (Qiagen). RU5 per cell was measured as described [48].

### Statistics

Statistical tests, including Wilcoxon, Mann-Whitney and Linear Regression tests were performed using GraphPad Prism software (GraphPad software, Inc., San Diego, CA). A p value < 0.05 was considered significant.
