# Correction: Gag-Positive Reservoir Cells Are Susceptible to HIV-Specific Cytotoxic T Lymphocyte Mediated Clearance *In Vitro* and Can Be Detected *In Vivo*

**DOI:** 10.1371/annotation/3aa92c3d-b6dd-4c6e-8cee-9587ce80a9c9

**Published:** 2013-09-13

**Authors:** Erin H. Graf, Matthew J. Pace, Bennett A. Peterson, Lindsay J. Lynch, Steve B. Chukwulebe, Angela M. Mexas, Farida Shaheen, Jeffrey N. Martin, Steven G. Deeks, Mark Connors, Stephen A. Migueles, Una O’Doherty

This article has been republished because an incorrect, earlier version of the manuscript was published originally. 

